# Integrating Chemo- and Bioinformatics with *In Vitro* Biological Assays to Discover Potential ACE2 and Mpro Inhibitors against SARS-CoV‑2

**DOI:** 10.1021/acs.jcim.5c01056

**Published:** 2025-07-25

**Authors:** Ryan S. Ramos, João S. N. de Souza, Mariana H. Chaves, Joaquín M. Campos, Willyenne M. Dantas, Lindomar J. Pena, Maracy L. D. S. Andrade, Cleydson B. R. Santos

**Affiliations:** † Graduate Program in Biotechnology and Biodiversity-Network BIONORTE, Federal University of Amapá, Macapá 68903-419, Amapá, Brazil; ‡ Laboratory of Modeling and Computational Chemistry, Department of Biological and Health Sciences, Federal University of Amapá, 68902-280 Macapá, AP, Brazil; § Chemistry Department, 67823 Federal University of Piauí, Campus Universitário Ministro Petrônio Portela, Av. Nossa Senhora de Fátima, Bairro Ininga, CEP: 64.049-550 Teresina, PI, Brazil; ∥ Department of Pharmaceutical and Organic Chemistry, Faculty of Pharmacy, Campus of Cartuja, 16741 University of Granada, 18071 Granada, Spain; ⊥ Department of Chemistry, Federal Rural University of Pernambuco, Recife 52171-900, Brazil; # Department of Virology, Aggeu Magalhães Institute (IAM), 37903 Oswaldo Cruz Foundation (Fiocruz), Recife 50670-420, Brazil

## Abstract

The study aims to identify potential SARS-CoV-2 inhibitors and investigate the mechanism of action on the viral ACE2 receptor and main protease (Mpro), using chemo- and bioinformatics approaches. Ligand-based virtual screening was performed in the Molport database (∼4.79 million compounds), and after applying physicochemical filters, 313 molecules with characteristics such as hydroxychloroquine were obtained. After obtaining bioactive conformations, the molecular structures were subjected to the study of pharmacokinetic predictions, in which 106 molecules presented properties for oral bioavailability, penetration of the BBB, PPB, and solubility (average). The toxicological property predictions proved plausible for the molecules, as they did not present warnings of hepatotoxicity, mutagenicity, potential risk of carcinogenicity, and LC_50_ and LD_50_ values higher than the controls. Subsequently, 81 structures were subjected to a molecular docking study of ACE2 receptor/Spike and Mpro. In the ACE2 receptor, four (4) ligands showed high binding affinity value, in which the molecule MolPort-010-778-422 had the best Δ*G* value of −9.414 kcal/mol, followed by MolPort-009-093-282 with Δ*G* = −8.978 kcal/mol. In the Mpro receptor, four (4) ligands showed high binding affinity values compared to control 11b, with emphasis on molecule MolPort-005-766-143 with Δ*G* = −8.829 kcal/mol, followed by molecule MolPort-046-186-743. To study the antiviral effects of the molecules *in vitro*, TopHits8 molecules were tested against the SARS-CoV-2 virus. MolPort-010-778-422 had the best result on the screening and presented an IC_50_ of 8.9 nM.

## Graphical abstract

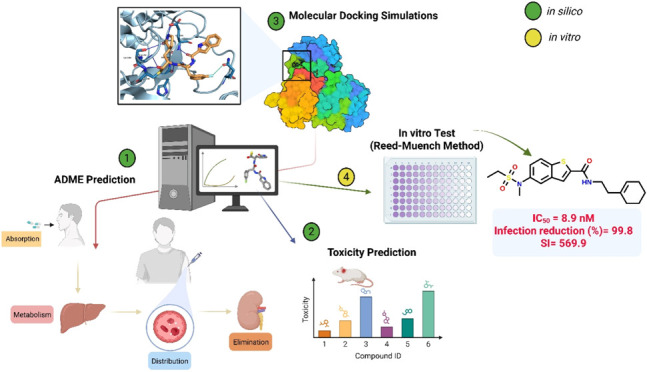

## Introduction

SARS-CoV-2, responsible for the COVID-19 pandemic, emerged in late 2019 in Wuhan, Hubei Province, China.^1–3^ Genetic analysis initially revealed a betacoronavirus as the causative agent. Symptoms of early infection were flu-like and included fever, cough, and myalgia but with a tendency to develop potentially fatal dyspnea and acute respiratory distress syndrome. Since then, viral infections have spread globally, significantly impacting public health and the global economy. According to the World Health Organization (WHO) (https://www.who.int/publications/m/item/covid-19-epidemiological-update-edition-172), as of 13 October 2024, over 776 million confirmed cases, more than 7 million deaths had been recorded due to COVID-19, with numbers varying depending on the availability of vaccination and the response of health systems in different countries.

SARS-CoV-2 infection is mediated by specific viral proteins that interact with receptors on the surface of human cells. The Angiotensin-Converting Enzyme 2 (ACE2) receptor is the main entry point for the virus, facilitating the binding and internalization of the viral particle through interaction with the spike protein.^4,5^ CoV’s encode a surface Spike glycoprotein, which binds to the host cell receptor and mediates viral entry. In betacoronaviruses, a single region of the Spike protein called the receptor-binding domain (RBD) mediates interaction with the host cell receptor.^6^ Studies have demonstrated advantages in the consistent use of peptides composed of D-amino acids for inhibiting the RBD-ACE2 interaction; two designs bind with affinity between 29 and 31 nM and inhibited SARS-CoV-2 infection in Vero cells with IC_50_ values of 5.76 and 6.56 μM.^6,7^ The interaction interface can be divided into three predominantly polar contact regions, resembling the structure of the SARS-CoV–ACE2 complex. In this conformation, an extended loop of the RBD interacts with the arc-shaped α1 helix of the proteolytic domain (PD) of ACE2, involving three main regions: N-terminal (cluster 1), central (cluster 2), and C-terminal (cluster 3). Additionally, helix α2 and loop 3–4 (connecting strands β3 and β4) of ACE2 contribute limited contacts. In cluster 1, located at the N-terminal end of helix α1, residues Gln498, Thr500, and Asn501 of the RBD form hydrogen bonds with Tyr41, Gln42, Lys353, and Arg357 of ACE2. The intermediate region (cluster 2) of the RBD loop establishes contact through residue Tyr453, with residue His34 of ACE2. Finally, in cluster 3, corresponding to the C-terminus of helix α1, residue Gln474 of the RBD interacts with Gln24 of ACE2, while Phe486 of the RBD establishes van der Waals interactions with Met82 of ACE2.^8^

During the initial phase of the COVID-19 pandemic (2019–2021), hydroxychloroquine (HCQ) and chloroquine (CQ) were rapidly prioritized as candidate antivirals due to their previously reported activity against SARS-CoV-1 and their ability to inhibit SARS-CoV-2 replication *in vitro*, particularly in Vero E6 cells.^9,10^ These findings prompted global clinical investigations and their inclusion in large-scale trials, such as the WHO Solidarity Trial and the UK RECOVERY Trial.^11,12^ Although later clinical data demonstrated limited efficacy and increased risk of adverse events, particularly cardiac toxicity, HCQ remained a key experimental control *in vitro* for antiviral screening platforms throughout the early pandemic.

After entry into the host cell, SARS-CoV-2 genomic RNA directly associates with ribosomes, promoting the translation of two large polyproteins, which are subsequently processed through proteolysis into several components required for packaging new virions. This proteolytic processing is mediated by two specific enzymes: the coronavirus main protease (Mpro) and the papain-like protease (PLpro).^13,14^ Furthermore, the Main Protease (Mpro), responsible for processing viral proteins during replication, is an important therapeutic target. Several SARS-CoV-2 variants, including α, Delta, Omicron, and others, have presented mutations that may impact their transmissibility, virulence, and ability to evade immune responses, complicating pandemic control efforts.^15,16^ The first crystal structure of Mpro in complex with the peptide inhibitor N3 was determined by Jin et al.^17^ These additional crystal structures of two rationally designed covalent peptide inhibitors, 11a and 11b were later reported by Dai et al.^14,18^

In response to the need for new therapeutic options, alternative treatments were investigated, such as the use of monoclonal antibodies, protease inhibitors, and repurposed drugs from other indications, such as antivirals for HIV and hepatitis C.^19,20^ Approaches that combine the use of corticosteroids, cytokine inhibitors, and antiviral therapies aim not only to suppress viral replication but also to modulate the exacerbated immune response associated with severe forms of COVID-19.^21,22^ However, side effects and the development of viral resistance are persistent concerns, driving the search for new treatments.^23^

Chemo- and bioinformatics have played a central role in the search for novel molecules to inhibit therapeutic targets, such as ACE2 and Mpro. Virtual screening methods, molecular docking, and molecular dynamics simulations are used to predict potential interactions between candidate compounds and viral proteins, allowing the prioritization of compounds for synthesis and experimental testing.^24–26^ These studies have identified promising ligands that inhibit binding of SARS-CoV-2 to ACE2 or block the catalytic activity of Mpro, providing innovative and more targeted therapeutic strategies to combat COVID-19. Therefore, this study aims to identify potential inhibitors and investigate the mechanism of action on the SARS-CoV-2 ACE2 receptor and main protease (Mpro), using the study of chemo- and bioinformatics.

## Results

### Predictions of Pharmacokinetic and Toxicological Properties

Compared to the control groups, the bioactive conformations of 313 molecular structures were analyzed through pharmacokinetic predictions to classify them based on the optimal oral bioavailability profile across five descriptors. Parameters related to absorption, distribution, metabolism, and excretion (ADME) play a fundamental role in the development of new drugs, since prior analysis of pharmacokinetic properties increases the likelihood of approval by regulatory agencies in the early stages of clinical trials. Thus, the pharmacokinetic descriptors for the TopHits8 molecules were determined, which stood out for presenting high oral bioavailability, ability to cross the Blood-Brain Barrier (BBB), satisfactory Human Intestinal Absorption (HIA), high Plasma Protein Binding (PPB) rate and good solubility in aqueous medium, as shown in Figure 1 and Table 1.

**Figure 1:**
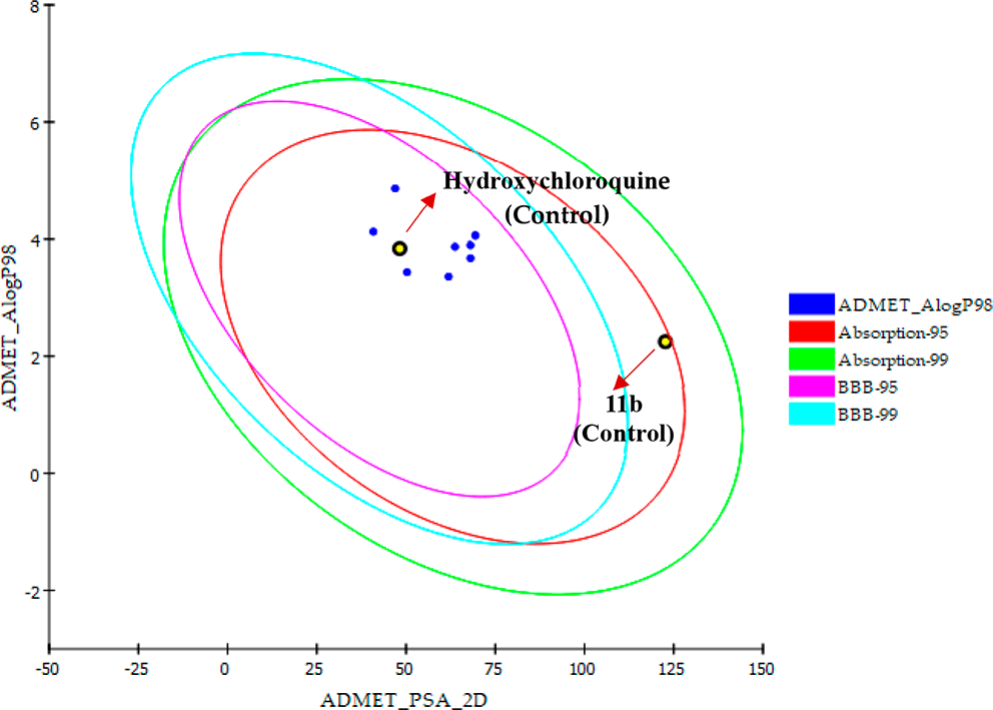
Pharmacokinetic property profile of TopHit8 molecules structures for oral bioavailability (AlogP98vsPSA).

**Table 1:** Oral Bioavailability of TopHits8 Molecules^a^

molecules	PPB	hepatotoxic	CYP2D6	solubility	BBB	HIA
hydroxychloroquine	false	true	true	3	1	0
11b	true	true	false	2	4	0
MolPort-010-778-422	true	true	false	2	2	0
MolPort-009-093-282	true	true	false	2	1	0
MolPort-004-953-083	true	true	false	2	1	0
MolPort-006-606-981	true	true	false	2	1	0
MolPort-045-920-671	true	true	false	2	1	0
MolPort-046-186-743	true	true	false	2	2	0
MolPort-002-201-288	true	true	false	2	2	0
MolPort-005-766-143	true	true	false	2	2	0

a BBB, blood–brain barrier: 0 (Very high penetrant); 1 (High); 2 (Medium); 3 (Low); 4 (very low); HIA, Human Intestinal Absorption (acceptable range: range is 0–2, where 0 is a good absorption); Aqueous solubility, (acceptable range: range is 0–3, where 3 is a good solubility); Cytochrome P450 (CYP450) 2D6 inhibition (false@@—noninhibitor, true—inhibitor); PPB, plasma–protein binding (false—does not bind to plasma proteins, true—binds to plasma proteins). Hydroxychloroquine (control); 11b (control).

Before carrying out ligand–receptor interaction studies, prior knowledge of the properties of a molecule is essential, including its physicochemical characteristics, similarity with known drugs, bioactivity scores, and oral bioavailability, as well as ADMET parameters and toxicity profiles (Radhakrishnan et al., 2023). All of these pharmacokinetic and toxicological parameters must follow the standardized Lipinski rule (Rule of 5), which is available for the determination of such properties. Regarding the ADMET analysis, among the 313 molecules, it is observed that 8 (TopHits8) stands out in comparison to the control groups for presenting a good pharmacokinetic profile, that is, ease of binding to plasma proteins, good water solubility, intestinal absorption and high penetration capacity in the blood-brain barrier (Table 1).

Toxicological studies in animals demonstrate that hydroxychloroquine is approximately 40% less toxic than chloroquine. The results of the toxicological predictions for Tophits8 molecules can be seen in Tables 2, Figure 2, and the Supporting Information for extended information.

**Figure 2:**
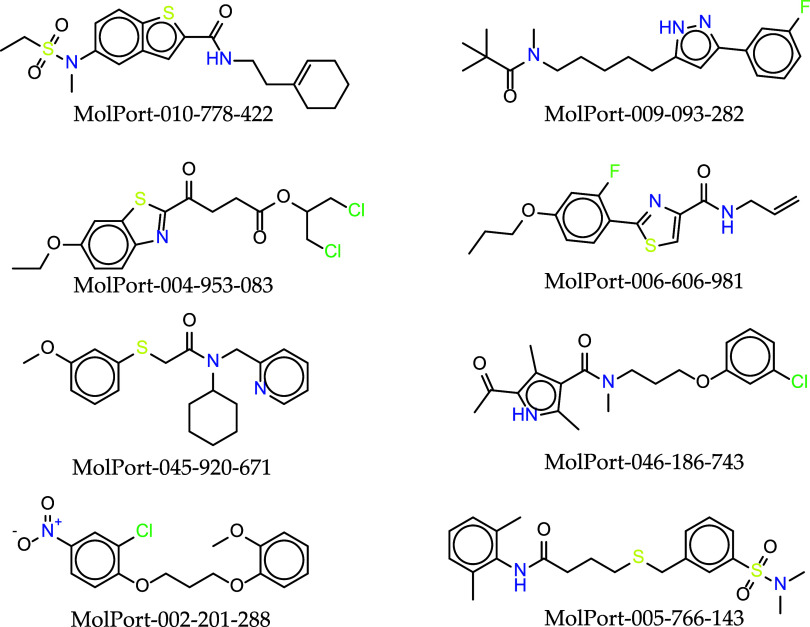
Molecular structure (2D) of the TopHits8 compounds that presented low risk of toxicity.

**Table 2:** Computational Evaluation of Toxicological Parameters, Toxicity Risk, and Tolerable Carcinogenic Doses of TopHit8 in Animal Models^a^

	computational toxicological parameters						prediction od toxicity risk					tolerated dose of carcinogenic potency		
molecules							*Pimephales promelas*	*Daphnia magna*	rat			rat	mouse	rat
	female/male								oral	chronic	innalation	(body weight/day)		
	mouse	rat	ames mutagenicity	skin irritancy	sensitization	ocular irritation	LC_50_ (g/L)	EC_50_ (mg/L)	LD_50_ (g/kg)	LOAEL (g/kg)	LC_50_ (mg/m^3^/h)	TD_50_ (mg/kg)		RMTD (g/kg)
hydroxychloroquine	C–	C–	M+	–	–	++++	0.009	11.40	1.068	0.062	24,400.2	19.64	12.73	0.016
11b	C–	C–	M-	–	–	+++	0.001	0.22	1.465	0.092	1,511.87	1.92	28.74	0.093
MolPort-010-778-422	C	C–	M-	++	–	+++	2.56e-05	0.32	0.853	0.007	2,230.09	564.49	16.00	0.103
MolPort-009-093-282	C–	C–	M-	++	–	–	0.001	0.72	0.138	0.065	8,395.07	3.020	27.90	0.392
MolPort-004-953-083	C–	C	M-	–	–	++	0.0001	0.30	0.130	0.017	5,966.61	8.835	62.10	0.019
MolPort-006-606-981	C–	C–	M+	–	++++	++	0.0003	2.52	0.392	0.011	69,180	105.85	74.81	0.004
MolPort-045-920-671	C–	C–	M-	–	–	++++	0.0003	0.20	1.571	0.027	3,824.72	33.24	44.90	0.022
MolPort-046-186-743	C–	C–	M-	–	–	+++	0.003	2.37	0.117	0.051	1,627.24	11.08	115.50	0.051
MolPort-002-201-288	C–	C–	M-	++	++++	++	0.001	0.11	3.201	0.028	2,795.87	2.64	321.30	0.0002
MolPort-005-766-143	C–	C–	M-	–	+	+++	0.001	0.33	6.877	0.070	771.57	142.91	45.80	0.736

a MF: Mouse Female; RF: Rat Female; AM: Ames Mutagenicity; SI: Skin Irritancy; SS: Skin Sensitization; OI: Ocular Irritancy; OR: Oral Rate; LD_50_: Median lethal dose; FM: Fathead Minnow (Short-term toxicity to fish *Pimephales promelas*); LC_50_: Exposure concentration of a toxic substance lethal to half of the animals tested; DM: *Daphnia magna*; EC_50_: Half-maximal effective concentration (effective concentration of a substance causing adverse effects in 50% of the population tested—*Daphnia magna*); RCL: Rat Chronic LOAEL (lowest level of adverse effect observed); RI: Rat Inhalation; RMTD: Rat Maximum Tolerated Dose; TD_50_: Carcinogenic potency value; - (None); C (Single Carcinogen); C+ (Multi Carcinogen); C- (Non Carcinogen); M- (Non Mutagen); M+ (Mutagen); + (Weak); + + (Mild); + ++ (Moderate); + +++ (Severe)..

### Molecular Docking for ACE2 and Main Protease (Mpro) Receptor

Potential molecules present interactions at the RBD binding site. The molecular docking poses can be observed in Figure 3. It is worth noting that there are 14 shared amino acid positions used by both RBDs for the interaction with ACE2. Among these residues, eight show identity between the two RBDs, namely: Tyr449-Tyr436, Tyr453-Tyr440, Asn487-Asn473, Tyr489-Tyr475, Gly496-Gly482, Thr500-Thr486, Gly502-Gly488, and Tyr505-Tyr491, corresponding to SARS-CoV-2 and SARS-CoV, respectively.^27,28^

**Figure 3:**
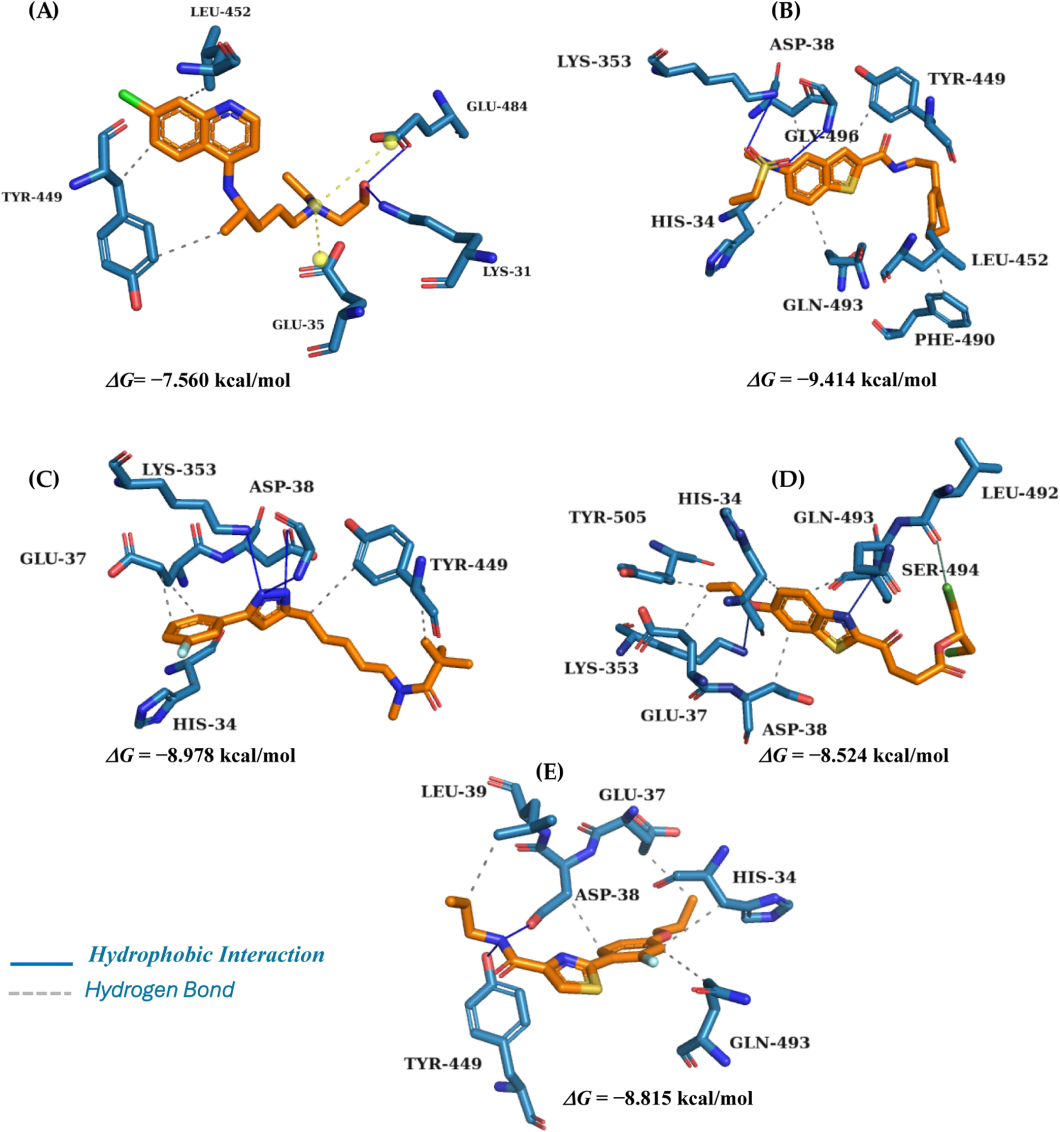
Interaction map representation of potential ligands: (A) Hydroxychloroquine, (B) MolPort-010–778–422, (C) MolPort-009–093–282, (D) MolPort-004–953–083, and (E) MolPort-006–606–981 in the Spike RBD active site.

The results obtained in the validation of the molecular docking simulation protocols were considered satisfactory, since the relative molecular overlay (pose, orientation, and torsion) between the crystallographic ligand (control—experimental model) and the redocked ligand (docking pose—theoretical model) showed high similarity. The recovery of the pose of the Mpro complex (11b, Figure 4A) allowed the validation of the molecular docking protocols, obtaining a root-mean-square deviation (RMSD) of 1.84Å—a value that represents the average distance between the atoms of the two conformations compared—and a binding free energy (Δ*G*) of −8.905 kcal/mol (Figure 1S). According to the literature, RMSD values equal to or lower than 2Å indicate that the docking protocol presents satisfactory quality, since it reproduces with high similarity the experimentally observed structure.^29–33^

**Figure 4:**
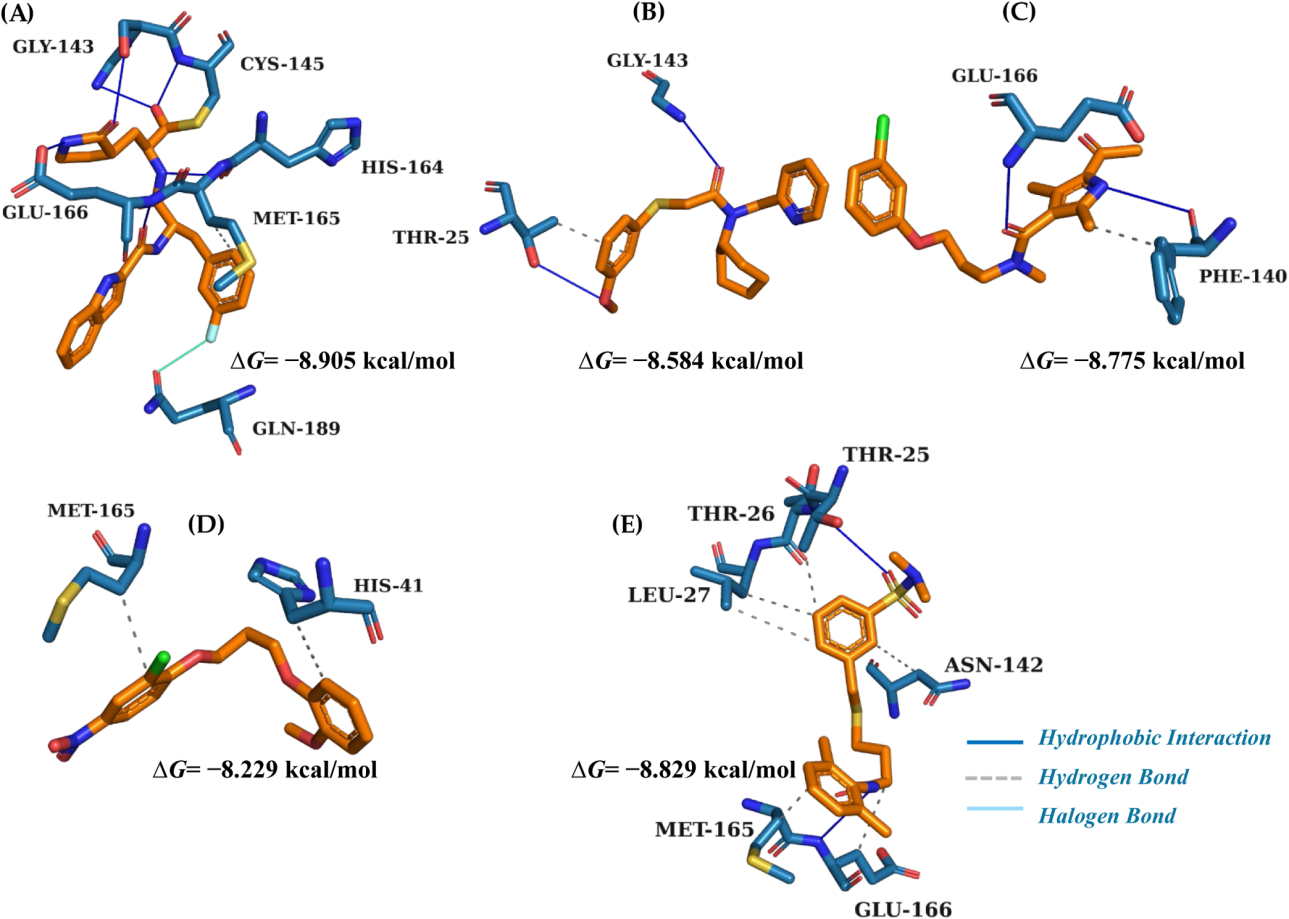
Interaction map representation of potential ligands (A) 11b, (B) MolPort-045–920–671, (C) MolPort-046–186–743, (D) MolPort-002–201–288, and (E) MolPort-005–766–143 in the Mpro receptor.

The molecules selected in the molecular docking study presented binding affinity values ranging from −8.229 to −8.829 kcal/mol, about the 11b control at −8.905 kcal/mol. The interactions in the binding site of the potential ligands MolPort-045–920–671 (Figure 4B), MolPort-046–186–743 (Figure 4C), and MolPort-002–201–288 (Figure 4D) were like 11b with the amino acid residues located in the α-helix with His41 and in the β-sheet with Gly143, Glu166, and Met165. The ligand (Figure 4E) MolPort-005–766–143 with the best binding affinity value of −8.829 kcal/mol showed main interactions like control 11b. The difference in binding affinity for the control was ±0.076. According to the literature, the interactions with the key amino acid residues are in the β-sheet between residues Leu27, Met165, and Glu166.

### Determination of CC_20_ and CC_50_ Values from Cytotoxicity Analysis on Vero Cells

After the data obtained from the cytotoxicity assay in Vero cells were collected, statistical analysis was performed to determine the CC_20_ and CC_50_ values, which indicates the concentration of the compound that reduces cell viability by 20% or 50%, respectively. The CC_20_ value represents the concentration at which 80% of the cells remain viable and was empirically defined as the maximum concentration for antiviral testing. For CC_20_, the value ranged from 2.258 to 29.30 μM; for CC_50_, the value ranged from 5.072 to 47.11 μM (Table 3). Based on the CC_20_ value used in the screening test, it is noteworthy that MolPort-010-778-422 was the most cytotoxic, while MolPort-004-953-083 was the least toxic in the series (Figure 5 and Table 3).

**Figure 5:**
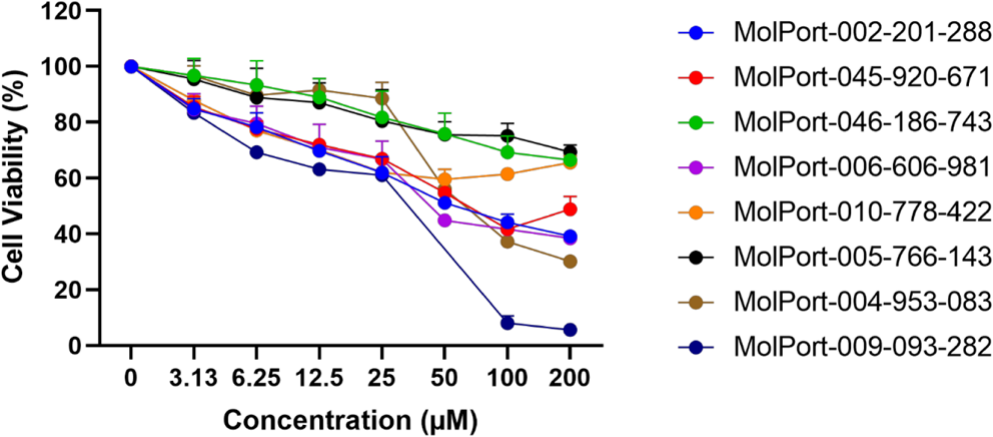
Cytotoxicity analysis of TopHits8 compounds tested on Vero cells. Compounds were incubated in a 96-well microplate with 1 × 10^4^ cells/well at concentrations ranging from 200 to 3.13 μM. After 72 h at 37 °C and 5% CO_2_ atmosphere, viable cells were measured using the MTT method. Nonlinear regression analysis was performed to calculate the CC_20_ and CC_50_ concentration values for each compound.

**Table 3:** Results of Cytotoxicity Analysis in Vero Cells^a^ ^c^.

molecules	CC_20_ ^b^ (μM)	CC_50_ ^b^ (μM)
MolPort-002-201-288	3.031	20.63
MolPort-045-920-671	2.772	16.12
MolPort-046-186-743	7.583	27.57
MolPort-006-606-981	3.964	19.37
MolPort-010-778-422	2.258	5.072
MolPort-005-766-143	3.532	15.74
MolPort-004-953-083	29.30	45.45
MolPort-009-093-282	9.418	47.11

a CC_20_ and CC_50_ values of the TopHits8 compounds.

b CC_20_ (20% cytotoxic concentration) refers to compound concentration that causes a 20% reduction in cell viability.

c CC_50_ (50% cytotoxic concentration) refers to compound concentration that causes a 50% reduction in cell viability.

### *In Vitro* Activity of TopHits8 Molecules against SARS-CoV-2 Virus

Following the determination of CC_20_ and CC_50_ values, a screening was performed with the TopHits8 compounds at the CC_20_ concentration against infectious SARS-CoV-2. All compounds demonstrated significant results after statistical analysis (Figure 6), with emphasis on the compound MolPort-010-778-422, which was able to reduce the viral titer by 5.6 log after TCID_50_ analysis of the samples (Figure 7 and Table 4).

**Figure 6:**
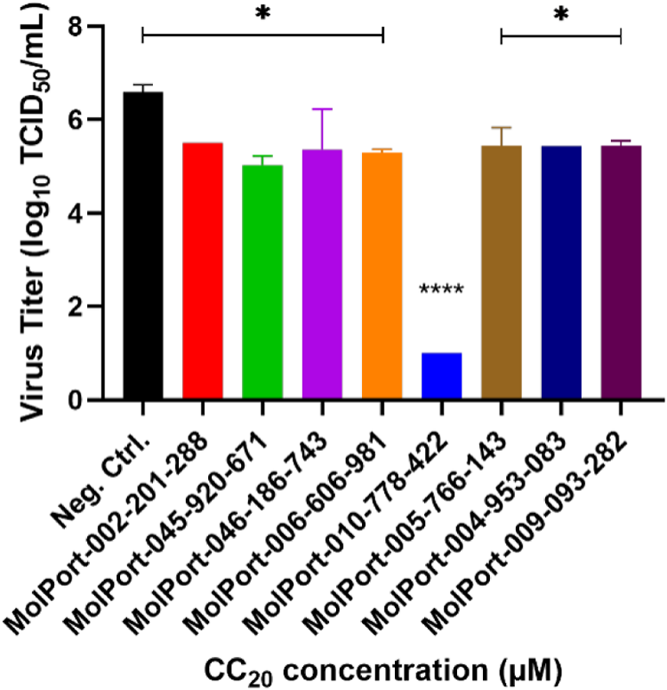
Antiviral activity of TopHits8 compounds against the SARS-CoV-2 virus. Vero cells were plated at 2.5 × 10^4^ cells/well and infected with SARS-CoV-2 at an MOI of 0.1. After 2 h of incubation, the compounds were added at the CC_20_ concentration. Following 72 h of incubation, the supernatant was collected and titrated using the TCID_50_ method. Statistical analysis was performed using the ANOVA method and Dunnett’s test, with *P* < 0.05 considered significant.

**Figure 7:**
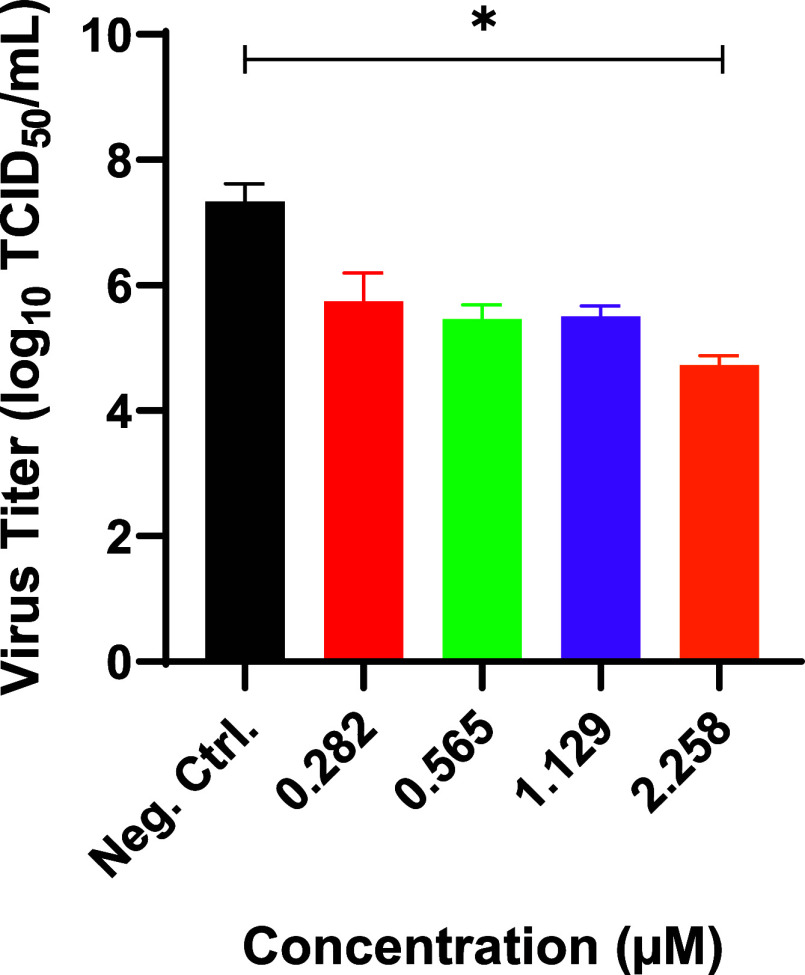
Dose-dependent antiviral activity of the MolPort-010–778–422 molecule against SARS-CoV-2. Vero cells were plated at 2.5 × 10^4^ cells/well and infected with SARS-CoV-2 at an MOI of 0.1. After 2 h of incubation, MolPort-010–778–422 was added to the plate at concentrations 2.258, 1.129, 0.565, and 0.282 μM. Following 72 h of incubation, the supernatant was collected and titrated using the TCID_50_ method. Statistical analysis was performed using nonlinear regression analysis.

**Table 4:** Results of MolPort-010-778-422 Molecule of Analysis against SARS-CoV-2

molecule	IC_50_ ^a^ (μM)	% infection reduction	log reduction	SI^b^
MolPort-010-778-422	0.0089	99.8	5.6	569.9

a Concentration of drug required for 50% inhibition.

b Selectivity index.

As the best hit molecule was identified, the IC_50_ of MolPort-010–778–422 molecule was determined using 1:2 serial dilutions from the CC_20_ concentration (CC_20_/2, CC_20_/4, and CC_20_/8), corresponding to the concentrations 2.258, 1.129, 0.565, and 0.282 μM. Following nonlinear regression analysis, the IC_50_ value for the compound was determined to be 0.0089 μM (8.9 nM). The selectivity index was calculated to be 569.9, and the percentage of infection reached 99.8% (Figure 7 and Table 4), characterizing the MolPort-010–778–422 as an excellent prototype candidate for antiviral activity against the SARS-CoV-2 virus.

## Discussion

A hepatotoxicity alert was observed in all molecular structures, as is expected to occur in humans in a dose-dependent manner.^34^ However, hydrochloroquine is a drug with proven antimalarial activity and has not been associated with significant elevations of serum enzymes during the therapy of rheumatological diseases. Both chloroquine and hydroxychloroquine are metabolized in the liver by cytochrome P450 enzymes.^35,36^ Preexisting liver conditions, such as hepatitis, alcoholism, or concomitant use of other drugs metabolized by the same P450 isoenzymes, may alter the metabolism of these drugs.^36,37^

According to data from Porta et al. (2020), the minimum lethal dose of chloroquine is estimated to be between 30 and 50 mg/kg. Total blood concentrations of 2.5 μg/L or higher are considered lethal, and significant hydroxychloroquine toxicity has been reported in patients with plasma levels ranging from 2.05 to 18.16 μmol/L (640 μg to 6100 μg/L), with fatalities showing post-mortem blood concentrations of 142.89 μmol/L (48,000 μg/L) and 309.62 μmol/L (104,000 μg/L).^38^

Chloroquine and hydroxychloroquine are aminoquinoline derivatives widely used in the treatment of rheumatologic diseases as well as in malaria prophylaxis. Acute toxicity due to aminoquinolines is rare, and the contemporary literature on its management is limited. As with other toxicological emergencies, there is a lack of randomized clinical trials and systematic reviews that evaluate management approaches. The proposed utility of aminoquinolines in mitigating the severity of infection is hypothesized to result from preventing SARS-CoV-2 from binding to target receptors and inhibiting viral entry into host cells. Both chloroquine and hydroxychloroquine concentrate within endosomes, where they are thought to modulate organelle pH, inhibit the formation of autophagosomes, and impair the cleavage of the SARS-CoV-2 spike protein.^38^

Considering the wide contact surface between the RBD domain of the Spike protein and the ACE2 enzyme, to carry out molecular docking studies at this binding site, the grid box configuration was centered on the Cα atom of residue Gln493, located at the interaction interface between Spike and ACE2, as described by Ramos et al.^29,39^

Molecular docking simulations of the poses generated allowed us to observe that the potential ligands interact with the amino acid residues of the Spike RBD active site around the α-helix between the amino acid residues Tyr449-Phe486 and are comprised in the β-sheet between the amino acid residues Lys31-Leu39.^40–42^

In the ligands, it is possible to observe hydrogen bond interactions with the amino acid residues of His34, Glu37, Lys353, Tyr449, Leu452, Phe490, Gln493, and Tyr505; it is possible to observe hydrophobic interactions with many residues in Asp38, Leu452, Phe490, and Leu492, results that agree with studies in literature.

The interactions of the potential inhibitors with the amino acid residues Tyr449, Gln493, Ser494, and Tyr505 in the RBD domain of the Spike protein are consistent with those reported in the literature. The inhibitors with the best binding layers were (Figure 3B) MolPort-010–778–422 (Δ*G* = −9.414 kcal/mol), (Figure 3C) MolPort-009–093–282 (Δ*G* = −8.978 kcal/mol), (Figure 3D) MolPort-004–953–083 (Δ*G* = −8.524 kcal/mol) and (Figure 3E) MolPort-006–606–981 (Δ*G* = −8.815 kcal/mol), whose interactions demonstrated similarity with the control, especially for residues Glu35 and Ser494, which contributed to the increase in the binding interaction. Furthermore, additional interactions involving residues Asp38, Leu39, Tyr351, Tyr449, Phe490, Glu494, and Tyr505 were observed, aiding in the stabilization of the active site for the Spike protein interaction in the RBD domain.

Evidence from the literature indicates that the 3CLpro/Mpro binding site is in the pocket formed between domains I and II and is composed of a Cys-His catalytic dyad (Cys145 and His41). This active site is predominantly composed of hydrophobic residues, among which the following stand out: Tyr54, Met49, Met165, Phe140, Leu141, Cys145, Leu27, Pro168, Leu167, Ala191, Cys44, Leu50, and Met40. The interactions observed in the molecular docking study in control 11b (Figure 4A) are similar in molecules MolPort-045–920–671 (Figure 4B) and MolPort-002–201–288 (Figure 4D) in the active site of Mpro, and others are atypical in molecules MolPort-046–186–743 (Figure 4C).

Yang et al. evaluated the effect of chloroquine and hydroxychloroquine on Vero cells and determined the CC_50_ value under different conditions. Considering the 72 h condition without hypoxia evaluated by the study, chloroquine presented a CC_50_ of 92.35 μM and hydroxychloroquine of 56.19 μM.^43^ Studies conducted by Liu et al. reported CC_50_ values of 273.20 μM for chloroquine and 249.50 μM for hydroxychloroquine.^44^ The findings suggest that the 8 selected compounds exhibit higher cytotoxicity compared to chloroquine and hydroxychloroquine under similar experimental conditions, as evidenced by their CC_50_ values. This highlights the need for additional studies to assess their safety and mechanism of action.

In comparison with the positive controls, chloroquine and hydroxychloroquine, the MolPort-010–778–422 molecule stands out as an excellent candidate for a potential antiviral against SARS-CoV-2. The analysis by Liu et al. presents IC_50_ values for chloroquine and hydroxychloroquine ranging from 2.71 to 7.36 μM and from 4.51 to 12.96 μM, respectively, under various test conditions.^44^ The selectivity index (SI) for chloroquine ranged from 100.81 to 37.12, and for hydroxychloroquine, it ranged from 55.32 to 19.25, both values lower than those presented by the MolPort-010–778–422 molecule. Comparative analysis of *in vitro* data reveals that MolPort-010–778–422 outperforms nonpeptide SARS-CoV-2 Mpro inhibitors reported in recent literature. With an IC_50_ value of 8.9 nM and a 5.6 log reduction in viral titer in Vero cells, the compound demonstrated significantly more potent antiviral activity than the inhibitors described by Jin et al.,^45^ which presented IC_50_ between 4.64 and 11.16 μM and EC_50_ between 12.25 and 29.32 μM; by Yang et al.,^46^ with IC_50_ ranging from 0.69 to 2.05 μM and EC_50_ up to 8.52 μM; and by Yang et al.,^47^ who reported activity against resistant mutants with IC_50_ between 8.78 and 27.81 μM. The *in vitro* results against SARS-CoV-2 for the molecule are consistent with the *in silico* findings for MolPort-010–778–422, which demonstrated the highest binding affinity of −9.414 kcal/mol.

Comparative analysis with Dai et al.^48^ revealed that the most promising molecule in this study, MolPort-005–766–143, exhibited a binding free energy (Δ*G* = – 8.829 kcal/mol) against Mpro that is comparable to the reference inhibitor 11b (Δ*G* = −8.905 kcal/mol), described by Dai et al. using X-ray crystallography and biochemical assays (IC_50_ = 0.040 μM). Although this study did not include direct enzymatic assays for Mpro inhibition, the binding pose of MolPort-005–766–143 demonstrated key interactions with the catalytic dyad (His41 and Cys145) and surrounding residues (Glu166 and Met165), consistent with the pharmacophore of potent Mpro inhibitors. Furthermore, the compound MolPort-010–778–422, which showed the highest antiviral potency in Vero cells (IC_50_ = 8.9 nM; SI = 569.9), presented superior cellular efficacy when compared to reported Mpro inhibitors, including 11b and others with IC_50_ values ranging from 0.69 to 11.16 μM. These findings highlight the strong potential of the selected candidates and justify subsequent enzymatic validation to confirm specific Mpro inhibition, as performed in the reference study.

## Methods

### Obtaining, Designing, and Geometric Optimization of Chemical Structures

The selected molecular structures were obtained from studies by Ramos et al. of ligand-based virtual screening (pharmacophoric model) to identify new potential inhibitors and clarify the mechanism of action of SARS-CoV-2 for pharmacokinetic, toxicological, and ligand/receptor interaction.^39^ Molecular structures were drawn using the ChemDraw Ultra 15.1.0 software and saved in the MDL Molfile (.mol and .sdf) formats.^49^ Subsequently, energy minimization and geometric optimization of the three-dimensional (3D) structures were performed using the ACD/ChemSketch (v. 12.01) software, using the Molecular Mechanics (MM+) method, whose force field was initially parametrized based on the CHARMM protocol.^50–53^

### *In Silico* Evaluation of Pharmacokinetic and Toxicological Properties

Pharmacokinetic and toxicological predictions of the investigated molecules were performed using an *in silico* approach, on the Discovery Studio v16.1 software (BIOVIA, Dassault Systèmes, San Diego, CA).^54,55^ To estimate the pharmacokinetic properties (ADMET), the ADMET Descriptors module was employed, providing predictive parameters, such as Human Intestinal Absorption (HIA), permeability in Caco-2 and MDCK cell lines, blood–brain barrier penetration (Log BB), plasma protein binding (PPB), and potential inhibition of cytochrome P450 (CYP2D6). The calculations were performed using default software settings based on validated QSAR models, as described in recent studies.

In the toxicological analysis, the TOPKAT (Toxicity Prediction by Komputer Assisted Technology) module was used, which estimates safety parameters from the 2D structure of the molecule. Indicators such as carcinogenicity in rodents (TD_50_), mutagenicity (Ames test), and acute oral toxicity (LD_50_), in addition to potential effects of irritation and dermal sensitization, as well as ecotoxicological toxicity for *Daphnia magna* and *Pimephales promelas*, were considered.

### Study of Molecular Docking Simulations

Crystal structure of SARS-CoV-2 spike receptor-binding domain bound with ACE2 (*Homo sapiens* organism), PDB ID 6M0J, resolution of 2.45 Å, and elucidated by the X-ray diffraction method;^4^ Crystal structure of SARS-CoV-2 main protease in complex with an inhibitor 11b, witch PDB ID 6M0K and a resolution of 1.50 Å were downloaded in the Protein Data Bank (PDB) (https://www.rcsb.org/) in the format (.pdb).^48^ The hydroxychloroquine, ritonavir, lopinavir, and 11b ligands were used as positive controls, and all water molecules and cofactors were deleted. The assignment of protonation and tautomeric states of the ligands, as well as the removal of residual water molecules and protein cofactors, was performed using Discovery Studio v.16 (BIOVIA, Dassault Systèmes, 2008). Before molecular docking, the protein structures were carefully prepared by deleting nonessential heteroatoms and correcting missing side chains when necessary. Hydrogen atoms were added to the protein using the PROPKA algorithm with the protonation states adjusted according to the predicted p*K*_a_ values at physiological pH (7.4). The prepared ligands and protein files were then subjected to energy minimization using the CHARMm force field to ensure optimal geometry for subsequent docking simulations.^56^ Molecular docking simulations were performed using the DockThor server (https://dockthor.lncc.br/v2/).^57^ The default algorithm parameters were set as follows: (1) 12 docking runs for conformation generation; (2) 500,000 evaluations per docking run; and (3) a population size of 750 individuals. The quality of the protein–ligand docking score was assessed based on the root-mean-square deviation (RMSD) between the superimposition of the best-scoring binding mode and the experimental pose.

### Chemistry: General

The compounds MolPort-002–201–288, MolPort-045–920–671, MolPort-046–186–743, MolPort-006–606–981, MolPort-010–778–422, MolPort-005–766–143, MolPort-004–953–083, and MolPort-009–093–282 were prepared in large-scale custom synthesis by Molport Ltd. (US/EU) with purity ≥ 85%. All reagents were of analytical grade and were purchased from Sigma-Aldrich (St. Louis, MO).

### Cells and Virus

Vero cells, a mammalian cell line derived from the kidney of the African green monkey (*Cercopithecus aethiops*), were used for cytotoxicity and antiviral tests. The cells were cultured in DMEM supplemented with 2–10% fetal bovine serum (FBS) and 1% penicillin/streptomycin (pen/strep) under incubation conditions of 37 °C and 5% CO_2_. All culture procedures utilized products from Thermo Fisher Scientific. SARS-CoV-2 strain hCoV-19/Brazil/PE-FIOCRUZ-IAM4372/2021 (GISAID accession number EPI_ISL_5254314) was used in the antiviral tests. The viral stock was prepared in Vero cells and propagated under the same incubation conditions of 37 °C and 5% CO_2_. The end point dilution assay was employed to determine the TCID_50_/mL titer of the viral stock using the Reed-Muench method.^58,59^

### Cytotoxicity of TopHits8 Compounds in Vero Cells

Cytotoxicity test of the TopHits8 compounds was performed in Vero cells using 96-well plates, with 1 × 10^4^ cells/well, prepared 24 h in advance. The compounds were diluted in culture medium supplemented with 2% FBS and 1% penicillin/streptomycin.^60^ The concentrations used in the test were 200, 100, 50, 25, 12.5, 6.3, and 3.1 μM, maintaining a maximum DMSO concentration of 1%, as tolerated by the cells. After the compound dilutions were distributed on the microplate in quadruplicate, the plate was incubated for 72 h at 37 °C in a 5% CO_2_ atmosphere in an incubator. The MTT method (3-(4,5-dimethylthiazol-2-yl)-2,5-diphenyltetrazolium bromide) was used to determine the number of viable cells after 3–4 h of incubation with a 1 mg/mL solution (50 μL/well). The medium was then removed, and 100 μL/well of DMSO was added to solubilize the formazan crystals. Subsequently, the microplates were read using a BioTek ELx800 spectrophotometer (BioTek Instruments, Winooski, VT) at a wavelength of 570 nm. The cytotoxic concentration values for 20% of viable cells (CC_20_) and the median cytotoxic concentration (CC_50_) were determined through statistical analysis using Microsoft Office Excel (Microsoft, Redmond, WA) and GraphPad Prism v.9.0 software (GraphPad Software, Inc., San Diego, CA).

### *In Vitro* Evaluation of TopHits8 Compounds against SARS-CoV-2 Virus: Screening Test

The screening test was performed using the CC_20_ concentrations of each compound determined in the cytotoxicity test. Vero cells were plated in 48-well microplates at a density of 2.5 × 10^4^ cells/well, prepared 24 h in advance. The plate was infected with the virus at a multiplicity of infection (MOI) of 0.1, except for the cell control wells, which received only culture medium supplemented with 2% FBS and 1% penicillin/streptomycin (pen/strep). After 2 h of incubation at 37 °C in a 5% CO_2_ atmosphere, the wells were washed with DMEM, and the compounds were added in triplicate. The plate was then incubated for an additional 72 h. After this period, the supernatants were collected from the wells, stored at −80 °C, and subsequently titrated using the TCID_50_ methodology. Statistical analysis was performed using GraphPad Prism v.9.0 software (GraphPad Software, Inc., San Diego, CA).

### IC_50_ Determination of MolPort-010–778–422 Compound

To determine the median inhibitory concentration (IC_50_) value of the MolPort-010–778–422 compound, concentrations of 2.258, 1.129, 0.565, and 0.282 μM were used, with the CC_20_ concentration serving as the initial concentration. Vero cells were plated in 48-well microplates at a density of 2.5 × 10^4^ cells/well, prepared 24 h in advance. The plate was infected with the virus at a multiplicity of infection (MOI) of 0.1, except for the cell control wells, which received only culture medium supplemented with 2% FBS and 1% penicillin/streptomycin (pen/strep). After 2 h of incubation at 37 °C in a 5% CO_2_ atmosphere, the wells were washed with DMEM, and the compounds were added in triplicate. After this period, the supernatants were collected from the wells, stored at −80 °C, and subsequently titrated using the TCID_50_ methodology. Statistical analysis was performed using GraphPad Prism v.9.0 software (GraphPad Software, Inc., San Diego, CA).

### Titration of Stock Virus and Samples (TCID_50_ Method)

Samples from the experiment were diluted (1:10) with DMEM + 2% FBS and added in quadruplicate to the wells of a 96-well microplate (1 × 10^4^ cells/well of Vero cells), prepared 24 h in advance. Each dilution (100 μL/well) was added to the plate, which was then incubated for 72 h. Some columns of the plate were selected to serve as a cell control (culture medium only, no virus). The reading was performed using an inverted optical microscope, counting the positive wells by the presence of cytopathic effects. The determination of the sample titer using the Reed-Muench method was carried out with the aid of Microsoft Office Excel (Microsoft Office, Redmond, WA).

### Statistical Analysis

In the cytotoxicity test, the optical densities obtained from the spectrophotometer reading were converted into percentage of cell viability in the Microsoft Office Excel program (Microsoft Office, Redmond, WA) according to the following formula

$$\%\;\mathrm{viability}={\mathrm{OD}}_{\mathrm{sample}}\times 100/{\mathrm{OD}}_{\mathrm{cellular}\;\mathrm{control}}$$

The determination of the CC_20_ and CC_50_ values was obtained through nonlinear regression analysis by the values of the concentrations used in the test and the quadruplicate values of the viability. In the screening test, ANOVA and Dunnett tests were used, considering *p* ≤ 0.05 as the minimum significance value. The determination of the IC_50_ value was obtained by nonlinear analysis of the titration values of the supernatants in triplicate of the concentrations used in the test. All analyses were performed in the GraphPad Prism v.9.0 program (GraphPad Software, Inc., San Diego, CA).

## Conclusions

ADMET studies have demonstrated that most of the molecules possess favorable absorption properties and exhibit low acute toxicity levels. Molecular docking analyses confirmed the interaction of these molecules at the ACE2 active site, with MolPort-010–778–422 showing the highest binding affinity of −9.414 kcal/mol. Additionally, MolPort-005–766–143 with Δ*G* = – 8.829 kcal/mol to Mpro, indicating strong interactions. Cytotoxicity analysis identified MolPort-004–953–083 as the least toxic compound in the series, with a CC_20_ of 29.30 μM and a CC_50_ of 45.45 μM. In contrast, MolPort-010–778–422 was found to be the most toxic, with a CC_20_ of 2.258 μM and a CC_50_ of 5.072 μM. The screening test revealed MolPort-010–778–422 as the most promising compound, with an IC_50_ of 0.0089 μM, a selectivity index (SI) of 569.9, and a 99.8% infection reduction. Molecules with potential antiviral activity can be synthesized using green chemistry techniques and environmentally friendly solvents. The integration of *in silico* studies and *in vitro* biological assays represents a rational and modern pipeline for antiviral discovery. The use of open software and public databases (DockThor and MolPort) made the study reproducible and accessible to other research groups. This approach aims to enhance the understanding of the mechanisms of action of SARS-CoV-2 inhibitors agents, ultimately guiding *in vitro* and *in vivo* biological assays.

## Supplementary Material



## Data Availability

All data used to generate the results of this study, including those presented in the main manuscript and the Supporting Information, are available in the Zenodo repository under the accession number https://zenodo.org/records/15365014.

## Notes

RMSD representation of the crystallographic ligand (green) and best docking pose (cyan) in the 11b (Figure S1) (DOCX)

## Abbreviations

ACE2: angiotensin-converting enzyme 2
ADMET: absorption, distribution, metabolism, excretion, and toxicity
AM: ames mutagenicity
BBB: blood–brain barrier
CC_20_: refers to compound concentration that causes a 20% reduction in cell viability
CC_50_: refers to compound concentration that causes a 50% reduction in cell viability
EC_50_: half-maximal effective concentration
HIA: human intestinal absorption
IC_50_: half-maximal inhibitory concentration
MF: mouse female
MOI: multiplicity of infection
Mpro: main protease
OI: ocular irritancy
OR: oral rate
PD: proteolytic domain
PLpro: papain-like protease
PPB: plasma–protein binding
RF: rat female
RBD: receptor-binding domain
RMSD: root-mean-square deviation
RMTD: rat maximum tolerated dose
SARS-CoV-2: severe acute respiratory syndrome coronavirus 2
SI: selectivity index
SS: skin sensitization
TCID_50_: fifty-percent tissue-culture-infective dose
WHO: World Health Organization
