# Supplementary material for: Integrating Chemo- and Bioinformatics with In Vitro Biological Assays to Discover Potential ACE2 and Mpro Inhibitors against SARS-CoV‑2
Source: J Chem Inf Model. 2025 Jul 25;65(15):8249–61. doi: 10.1021/acs.jcim.5c01056 (PMC12344719; doi:10.1021/acs.jcim.5c01056)
Supplement: Supplementary file 1 [file ci5c01056_si_001.docx]

**Supplementary Information for**

**Integrating chemo- and bioinformatics with *in vitro* biological assays to discover potential ACE2 and Mpro inhibitors against SARS-CoV-2**

Ryan S. Ramos ^a,b,*^, João S. N. de Souza ^c^, Mariana H. Chaves ^c^, Joaquín M. Campos^d^, Willyenne M. Dantas ^e,f^, Lindomar J. Pena ^f^, Maracy L. D. S. Andrade^b^ , Cleydson B. R. Santos ^a,b,c^*

^a^ Graduate Program in Biotechnology and Biodiversity-Network BIONORTE, Federal University of Amapá, Macapá, 68903-419 Amapá, Brazil; [ryanquimico@hotmail.com](mailto:ryanquimico@hotmail.com) (R.S.R)

^b^ Laboratory of Modeling and Computational Chemistry, Department of Biological and Health Sciences, Federal University of Amapá, 68902-280 Macapá, AP, Brazil;

^c^ Chemistry Department, Federal University of Piauí, Campus Universitário Ministro Petrônio Portela, Av. Nossa Senhora de Fátima, Bairro Ininga, CEP: 64.049-550, Teresina, PI, Brazil; [sammynery@ufpi.edu.br](mailto:sammynery@ufpi.edu.br) (J.S.NS.); [mariana@ufpi.edu.br](mailto:mariana@ufpi.edu.br) (M.H.C.)

^d^ Department of Pharmaceutical and Organic Chemistry, Faculty of Pharmacy, Campus of Cartuja, University of Granada, 18071 Granada, Spain;

^e^ Department of Chemistry, Federal Rural University of Pernambuco, Recife 52171-900, Brazil; [willyenne.dantas@ufrpe.br](mailto:willyenne.dantas@ufrpe.br) (W.M.D.);

^f^ Department of Virology, Aggeu Magalhães Institute (IAM), Oswaldo Cruz Foundation (Fiocruz), Recife 50670-420, Brazil; [lindomar.pena@fiocruz.br](mailto:lindomar.pena@fiocruz.br) (L.J.P.);

***** Correspondence: [breno@unifap.br](mailto:breno@unifap.br) (C.B.R.S)

**Table of Contents**

**1. Supplementary figures S1**

1. **Supplementary figures**


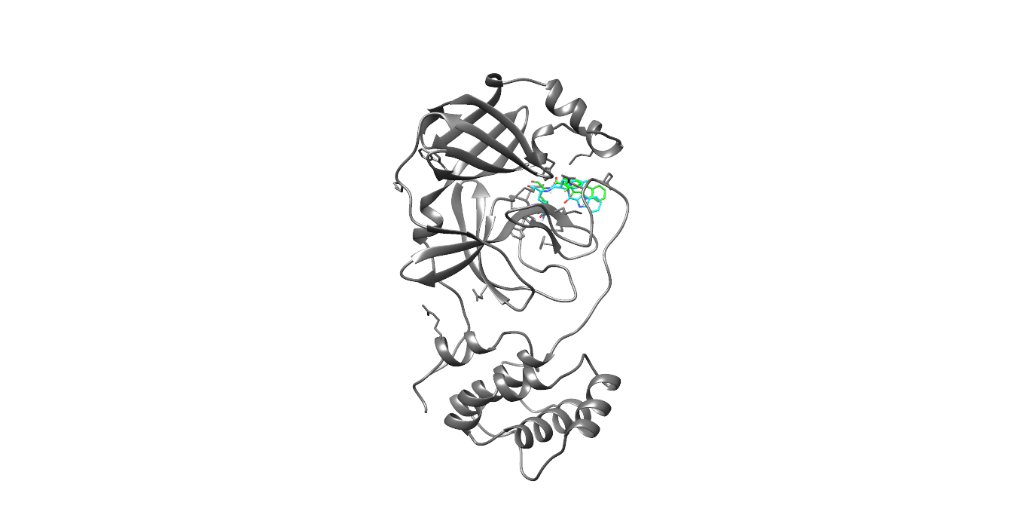

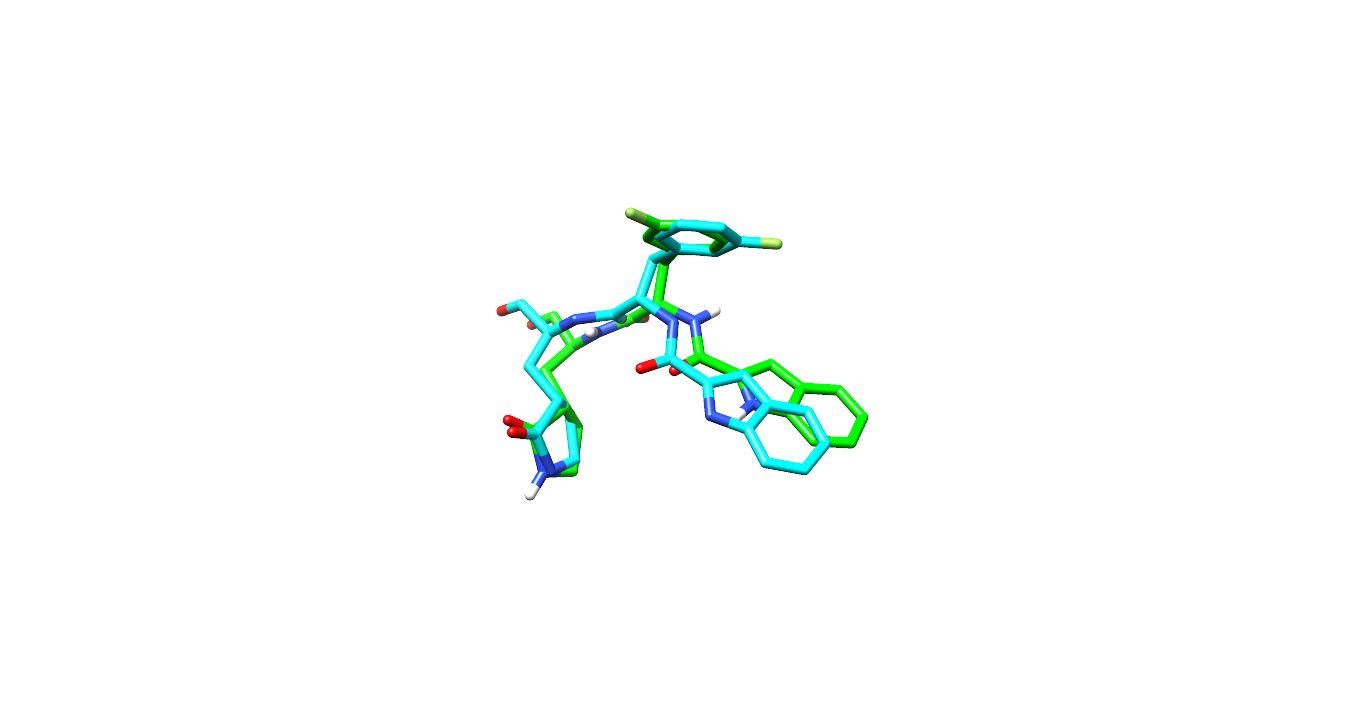


**RMSD= 1.84 Å**

***ΔG*= −8.905 kcal/mol**

**Figure S1.** RMSD representation of the crystallographic ligand (green) and best docking pose (cyan) in the 11b.
